# Adolescent obsessive–compulsive disorder presenting with internal dialogue: a case report

**DOI:** 10.3389/fpsyt.2026.1872475

**Published:** 2026-08-13

**Authors:** Doudou Yu, Xinchuan Lu, Ju Gao

**Affiliations:** 1 Department of Psychiatry, Yangzhou Wutaishan Hospital of Jiangsu, Yangzhou, Jiangsu, China; 2 Department of Psychiatry, The Affiliated Guangji Hospital of Soochow University, Suzhou, Jiangsu, China

**Keywords:** adolescent, differential diagnosis, internal dialogue, mental compulsions, obsessive–compulsive disorder

## Abstract

**Background:**

Obsessive-compulsive disorder (OCD) in adolescents may present with predominantly internal cognitive symptoms rather than overt rituals. Intrusive thoughts experienced as an inner dialogue may resemble psychotic symptoms, particularly if depressive symptoms and functional impairment are present. Such presentations may be difficult to recognize in clinical practice.

**Case presentation:**

This case report describes a 16-year-old girl with an eight-month history of school refusal and intrusive thoughts. She experienced repetitive mental reviewing and vivid internal dialogues but recognized them as her own thoughts. Depressive symptoms were also present. There were no fixed delusional beliefs or externally perceived hallucinations. The diagnosis was OCD with predominantly mental compulsions. Sertraline combined with psychotherapy produced minimal improvement. Given the limited response, the treatment plan was adjusted. Treatment with vortioxetine and low-dose aripiprazole was followed by symptom reduction and a gradual return to school.

**Conclusions:**

This case describes an atypical adolescent OCD presentation in which inner dialogic intrusive experiences and depressive symptoms obscured the underlying obsessive pathology. Careful phenomenological assessment is important to avoid misdiagnosis and delayed treatment in similar cases. Symptom improvement was observed after treatment modification, although the relative contribution of medication changes, psychotherapy, family support, symptom fluctuation, and school re-engagement cannot be determined in a single case.

## Introduction

Obsessive-compulsive disorder (OCD) in adolescence is increasingly recognized as a neurodevelopmental disorder characterized by significant phenotypic heterogeneity and dynamic development of symptoms (1). Although classical fear-avoidance and ritual-dominant presentations are well defined, a subset of adolescents primarily exhibit internalized, cognitively dominant obsessions that may be overlooked in routine clinical assessment (2). These phenotypes often involve limited overt compulsions, preserved insight, and prominent ruminative, self-referential mental activity, complicating differential diagnosis across OCD, mood disorders, and psychotic disorders (3).

The particularly elusive but clinically relevant phenotype is characterized by persistent ego-dystonic introspective ruminations, which are accompanied by vivid inner dialogue experiences. Affected adolescents may describe internally generated dialogic thoughts or inner speech-like experiences that superficially resemble hallucinations, while remaining self-generated, insight-preserved, and not externally localized (4, 5). From a neurocognitive point of view, these presentations may reflect atypical self- or reality monitoring of inner speech (6), together with impaired cognitive inhibition and altered interactions among frontoparietal, default-mode, and salience networks (7, 8). Clinically, this phenotype is often masked by secondary depressive syndromes, including emotional exhaustion, social withdrawal and school refusal (9), leading to diagnostic overshadowing and prolonged functional deterioration during a critical neurodevelopmental window (10).

Although selective serotonin reuptake inhibitors (SSRIs) remain the first-line pharmacotherapy for pediatric OCD, cognition-dominant and ruminative presentations may demonstrate partial or unstable response to serotonergic treatment alone (11). Vortioxetine, a multimodal serotonergic agent that inhibits the serotonin transporter and exerts both agonist and antagonist effects at multiple 5-HT receptors, has been associated with improvement in obsessive–compulsive, depressive, and anxiety symptoms in preliminary studies of SSRI-resistant OCD (12). In addition, low-dose aripiprazole may help regulate aberrant salience processing and frontostriatal gating through partial dopamine D_2_/D_3_ agonism and 5-HT_2_A antagonism, which could contribute to better control of intrusive cognitive activity (13).

To date, pharmacological strategies specifically addressing ruminative, internally focused OCD presentations in adolescents have been reported infrequently. Here we describe a 16-year-old girl with a depressive and self-referential obsessive presentation accompanied by severe school refusal, who showed symptomatic and functional improvement after treatment modification involving vortioxetine and subsequent low-dose aripiprazole augmentation. This case draws attention to a less well-described clinical presentation of adolescent OCD and suggests that treatment approaches informed by symptom profile may be useful in managing complex cases.

## Case presentation

A 16-year-old girl presented to our outpatient psychiatric unit with an 8-month history of persistent school refusal, emotional symptoms, and intrusive thoughts. She had previously maintained good academic performance and normal relationships with peers. There was no history of developmental delay, neurological illness, substance use, or a family history of psychosis or bipolar disorder.

### Symptom onset and evolution

Initially, the patient developed recurrent intrusive thoughts focused on the fear that she would be perceived negatively by her classmates and teachers. These thoughts were experienced as ego-dystonic, uncontrollable and highly stressful, causing marked distress and emotional exhaustion. After the usual classroom interactions, she would be preoccupied with questions about whether she had said something wrong, or if someone had misread her.

To reduce her anxiety, she began to engage in covert mental compulsion. She rehearsed the last few conversations in her mind, carefully checking her own words, tone, and facial expressions for any mistakes. She also engaged in repeated self-directed verbal reassurance, trying to convince herself that she had done nothing wrong. In addition, she imagined alternative responses she might have given, mentally rehearsing hypothetical conversations to avoid perceived social disapproval. At their peak, these obsessive ruminations and mental rituals occupied three to four hours per day and provided only brief relief before intrusive doubts returned.

In the months that followed, her ruminations intensified and developed into vivid internal dialogic experiences. She described these experiences as inner speech-like, first-person cognitive events occurring within her own mind rather than as voices heard from external space. They were not experienced as acoustic sounds and were not accompanied by a sensory quality of auditory hallucination. The content was involuntary, intrusive, and distressing. She described alternating internal perspectives: one urged her to continue monitoring, controlling, or avoiding thoughts, whereas another attempted to counteract or neutralize these impulses. The latter process often functioned as self-reassurance and became part of her covert mental rituals. She consistently recognized these experiences as self-generated, denied any external source or agent, and retained insight. Nevertheless, their vivid dialogue-like quality caused concern among family members and contributed to diagnostic uncertainty.

### Functional impact and affective symptoms

As symptoms progressed, the patient developed severe social withdrawal, a fear of crowded spaces, and a gradual withdrawal from school. School attendance dropped to about zero to one day a week and she often failed to complete her homework because of constant intrusive thoughts and mental checking rituals. At the same time, she experienced significant depressive symptoms, including persistent low mood, anhedonia, fatigue, decreased focus and hopelessness. These affective symptoms closely matched the severity of the obsessive ruminations and were perceived by the patient as secondary to a feeling of being trapped in her own thoughts.

### Mental status examination

The patient was alert and fully oriented. Speech was normal. No formal thought disorder was observed. Thought content was characterized by ego-dystonic intrusive ruminations and self-referential concerns. She reported internal dialogic experiences but consistently recognized them as self-generated mental events with preserved insight and intact reality testing. No externally perceived hallucinations or delusional beliefs were present. Judgment was intact, and affect was anxious and mildly depressed. Cognitive functioning appeared grossly preserved on clinical examination. No evidence of psychotic-spectrum disorder or cognitive disorganization was observed.

### Diagnostic assessment and clinical reasoning

The diagnosis of obsessive-compulsive disorder was clinically established according to the International Classification of Diseases, 10th Revision (ICD-10), based on symptom chronology, mental status examination, longitudinal clinical observation, and standardized symptom severity ratings. The core diagnostic evidence included recurrent intrusive and ego-dystonic thoughts, repetitive covert mental rituals, self-reassurance, active resistance, marked distress, and substantial time consumption. These symptoms occupied several hours per day at their peak and caused clinically significant functional impairment, including school refusal, reduced academic functioning, and social withdrawal. The baseline Y-BOCS score of 27 further supported clinically significant obsessive-compulsive symptom severity.

Obsessive-compulsive symptom severity was assessed using the clinician-rated Yale-Brown Obsessive Compulsive Scale (Y-BOCS), which was routinely used in our outpatient setting for older adolescents, while anxiety and depressive symptoms were assessed using the HAMA and HAMD-24, respectively. The ratings were completed by the treating psychiatrist during outpatient follow-up. No structured psychosis-risk interview, dissociation-specific scale, or structured suicidality scale was administered. Psychotic-spectrum symptoms, dissociative symptoms, manic or hypomanic symptoms, and suicidal ideation were reviewed clinically based on available outpatient records and follow-up interviews.

Phenomenologically, the internal dialogue was best conceptualized as an intrusive inner speech-like obsessive experience with associated self-reassurance rituals, rather than an externally localized auditory hallucination. It occurred within her own mental activity, lacked a clear acoustic or external sensory quality, was involuntary and distressing, and was consistently recognized as self-generated. On this basis, the internal dialogue was interpreted as an obsessive-compulsive phenomenon rather than a psychotic-spectrum symptom. There was no external attribution, fixed delusional conviction, formal thought disorder, or impaired reality testing.

Several differential diagnoses were considered. Social anxiety disorder was considered because the initial concerns involved fear of negative evaluation; however, the repetitive, intrusive, ritualized, and time-consuming nature of the thoughts exceeded typical social anxiety. Major depressive disorder was also considered because depressive symptoms were prominent, but these symptoms appeared secondary to persistent obsessive rumination rather than representing the primary syndrome. Trauma-related dissociation, autism spectrum traits, and bipolar spectrum disorder were less likely because there was no trauma history, dissociative episode, early developmental abnormality, restricted interests, manic or hypomanic symptoms, or antidepressant-emergent mood switching (**Table 1**).

**Table 1:** Phenomenological differentiation of the patient’s internal dialogue.

Feature	Patient’s presentation	Diagnostic implication
Ego-dystonicity	Unwanted, intrusive, and distressing	Supports obsessions rather than ordinary depressive rumination
Agency/ownership	Recognized as self-generated mental events	Against externally imposed voices or thought insertion
Externality	Experienced within her own mental activity, not external space	Against externally localized auditory hallucinations
Conviction	No fixed delusional belief; doubts fluctuated with anxiety	Against delusional conviction
Resistance	Attempts to suppress, counter, or neutralize the thoughts	Supports obsessive-compulsive process
Controllability	Poorly controllable but briefly relieved by mental checking or reassurance	Supports covert mental compulsions rather than psychotic hallucinations
Clinical function	Led to repeated mental checking, self-reassurance, and post-event replaying	More consistent with OCD than depression rumination alone

Taken together, the clinical presentation was most consistent with obsessive-compulsive disorder with predominantly mental compulsions, accompanied by secondary depressive and anxiety symptoms.

### Initial treatment

Initial treatment was initiated under the working diagnosis of adolescent depressive disorder with prominent anxiety and intrusive ruminations. Sertraline was titrated to 100 mg/day and maintained for approximately 8 weeks together with structured cognitive behavioral therapy. This duration was considered a reasonable initial trial for the depressive-anxiety presentation, although it did not represent a fully optimized SSRI trial for obsessive-compulsive disorder.

Psychotherapy initially focused on psychoeducation, behavioral activation, emotional regulation, and cognitive restructuring of negative self-evaluation. Because the obsessive-compulsive nature of the symptoms was not fully recognized at the beginning of treatment, a manualized exposure and response prevention protocol was not completed. The lack of structured ERP was subsequently recognized as a limitation of the initial treatment approach.

Because the patient continued to show persistent intrusive rumination, internal dialogue, and severe functional impairment, including school refusal, the treatment strategy was subsequently reconsidered.

### Treatment modification and outcome

Given the persistent functional impairment, prominent depressive symptoms, and severe intrusive rumination, the treatment plan was reconsidered after discussion with the patient and her family. Although further SSRI dose optimization, ERP intensification, and other guideline-supported strategies remained relevant options for OCD, vortioxetine 10 mg/day was selected to target the coexisting depressive symptoms and ruminative cognitive features.

Symptom changes were modest during the first month of vortioxetine monotherapy. Because obsessive rumination, internal dialogic experiences, and affective symptoms remained clinically significant, low-dose aripiprazole was introduced at month 1 and continued at 5 mg/day as adjunctive therapy. Gradual improvement was observed during subsequent follow-up; however, this temporal association should be interpreted cautiously given the uncontrolled single-case design.

**Figure 1:**
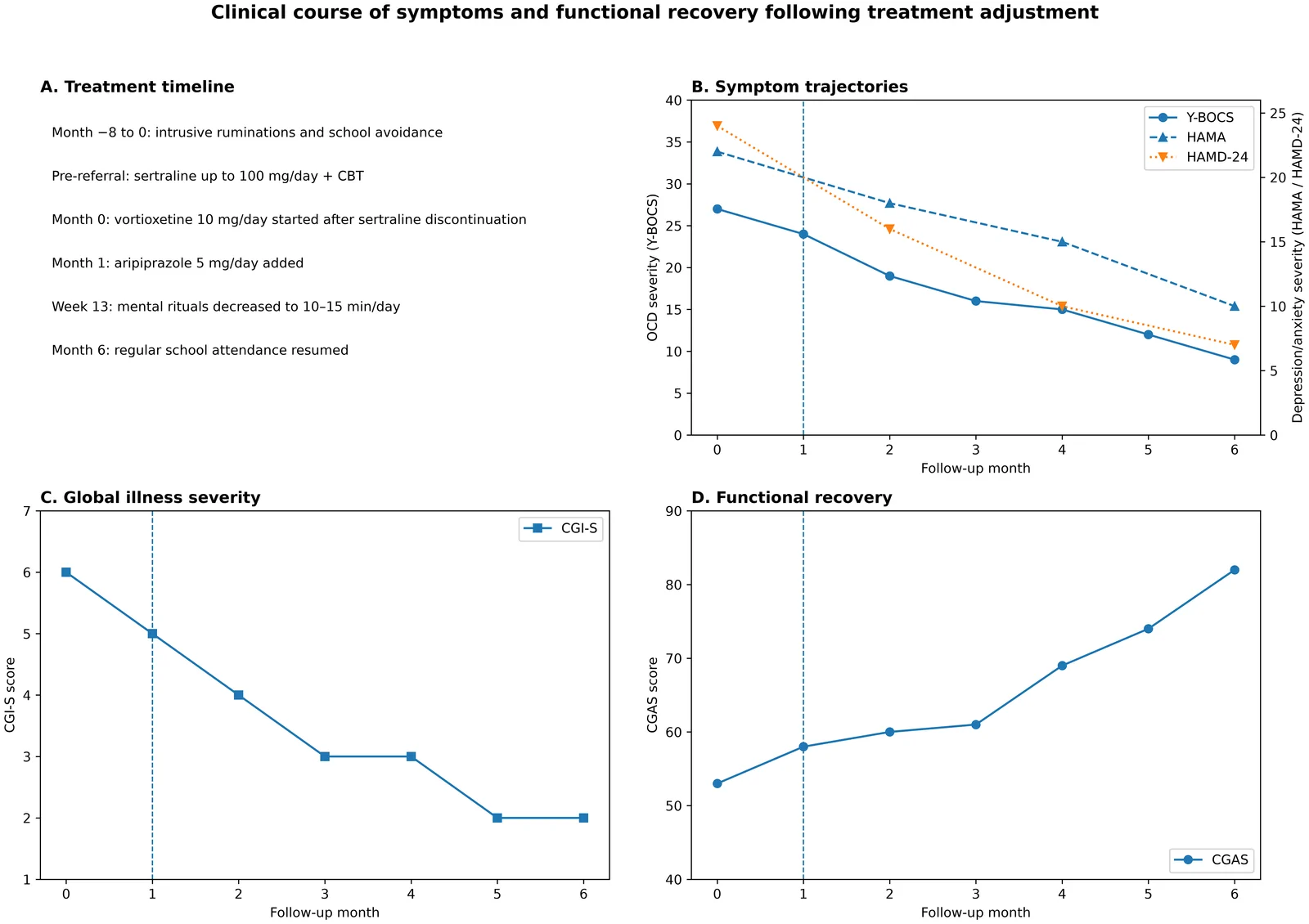
Clinical course of symptoms and functional recovery following treatment adjustment. **(A)** Treatment timeline showing symptom evolution and medication changes. Month 0 indicates the baseline assessment at the start of vortioxetine treatment after discontinuation of sertraline. Aripiprazole 5 mg/day was added at month 1. **(B)** Trajectories of obsessive-compulsive, anxiety, and depressive symptoms over six months, measured by the Yale-Brown Obsessive Compulsive Scale (Y-BOCS), Hamilton Anxiety Rating Scale (HAMA), and 24-item Hamilton Depression Rating Scale (HAMD-24). **(C)** Change in global illness severity measured by the Clinical Global Impression -Severity scale (CGI-S). **(D)** Functional recovery measured by the Children's Global Assessment Scale (CGAS). The vertical dashed line indicates the addition of aripiprazole at month 1. Lower scores on Y-BOCS, HAMA, HAMD-24, and CGI-S indicate clinical improvement, whereas higher CGAS scores indicate better global functioning.

By week 13, time spent on mental rituals had decreased to approximately 10–15 minutes per day and remained at this level through month 6. Her Yale-Brown Obsessive Compulsive Scale score decreased from 27 at baseline to 9 by month 6. The internal dialogic experiences gradually diminished and resolved. Depression symptoms also improved, with improvements in emotional stability, motivation, and concentration. Anxiety and depression symptoms showed parallel improvements, with Hamilton Anxiety Rating Scale (HAMA) scores decreasing from 22 to 10 and 24-item Hamilton Depression Rating Scale (HAMD-24) scores decreasing from 24 to 7. She successfully resumed regular school attendance and reintegrated into age-appropriate social functioning. Clinical Global Impression–Severity scale (CGI-S) scores decreased from 6 to 2, and Children’s Global Assessment Scale (CGAS) increased from 53 to 82 over six months (**Figure 1**).

Treatment adherence was assessed through outpatient follow-up interviews with the patient and her family, and no major adherence problems were reported. Tolerability and adverse events were monitored throughout follow-up, including sleep changes, sedation, akathisia or extrapyramidal symptoms, appetite or weight changes, prolactin-related symptoms, and suicidal ideation. Routine laboratory and physical safety monitoring, including complete blood count, blood biochemistry, and electrocardiography, was performed approximately every three months, with no clinically significant abnormalities identified. No clinically significant adverse events were reported or observed during follow-up.

### Patient perspective

During follow-up, the patient reported that the most meaningful change was the reduction in time spent on intrusive internal dialogue and mental checking. She described feeling less trapped by repetitive thoughts and was able to return to regular school attendance. Her family also reported improved emotional stability and greater confidence in daily social and academic functioning.

## Discussion

This case illustrates a less recognized and clinically challenging presentation of adolescent OCD, characterized by persistent intrusive self-referential thoughts, internal dialogue, secondary depressive symptoms, and functional impairment. Although the initial clinical manifestation was dominated by emotional exhaustion and anhedonia, extended evaluation led to a shift in diagnostic emphasis. These affective complaints were understood to arise from chronic obsessive rumination and covert mental compulsions, rather than from a primary mood disorder. The predominance of depressive symptoms obscured the underlying obsessive pathology and highlighted the significant risk of misdiagnosis and delayed initiation of appropriate treatment. As adolescent presentations are often dominated by internalizing distress, obsessive-compulsive features may be overlooked. In this case, the depressive predominance contributed to delayed recognition, illustrating how obsessive symptoms can be misinterpreted and treatment postponed.

A central clinical feature of this case was the presence of vivid internal dialogic experiences that mimicked psychotic-like symptoms. These experiences made it difficult to distinguish obsessional thinking and hallucination-like symptoms, creating a risk of misclassification as a psychotic-spectrum disorder (14). However, several key characteristics supported an obsessive rather than psychotic origin. The experiences remained ego-dystonic, were recognized as self-generated, and were associated with preserved insight and active resistance. The patient understood the dialogues as products of her own cognitive activity and attempted to suppress or counter them, indicating intact self-monitoring and reality testing. Such features are consistent with previous descriptions of intrusive thoughts that may acquire perceptual-like qualities yet remain under voluntary control, suggesting a distinct obsessive manifestation rather than prodromal psychosis (15, 16). Recognition of this pattern is particularly important in adolescents, in whom symptom articulation may be imprecise and mood symptoms often predominate (17).

The symptomatic and functional improvement observed after vortioxetine treatment and subsequent low-dose aripiprazole augmentation should be viewed as a clinical observation rather than evidence of treatment efficacy (18). The temporal sequence is clinically noteworthy; however, causal attribution is not possible in a single uncontrolled case. Medication adjustment, nonspecific psychotherapeutic support, family involvement, symptom fluctuation, and school re-engagement may all have contributed to the observed improvement.

The limited clinical response to sertraline in this patient should be interpreted cautiously. Cognitive inflexibility and altered function of frontoparietal and default-mode networks have been implicated in obsessive-compulsive disorder (19, 20). Sertraline, like other SSRIs, primarily inhibits presynaptic serotonin reuptake through the serotonin transporter and thereby enhances serotonergic neurotransmission (21). However, in this case, sertraline was used at 100 mg/day for approximately 8 weeks under an initial depressive-anxiety formulation, and the trial was not optimized for OCD. In addition, manualized ERP was not completed. Therefore, the limited early response should not be interpreted as evidence of SSRI inefficacy. For persistent OCD symptoms, established next-step considerations include optimization of SRI dose and duration, evidence-based psychotherapy, and pharmacological augmentation or alternative strategies in treatment-resistant cases (22, 23). The relevance of cognitive-network mechanisms or specific pharmacological mechanisms to the present case remains speculative.

Vortioxetine has a multimodal serotonergic profile involving serotonin transporter inhibition and modulation of several 5-HT receptor subtypes (24), and has been associated with cognitive effects in major depressive disorder (25). Low-dose aripiprazole augmentation has also been reported in some patients with SSRI-resistant OCD (26–28). In the present case, these pharmacological considerations provided a rationale for individualized treatment modification, particularly in the context of coexisting depressive symptoms and persistent intrusive rumination. Nevertheless, the improvement observed in this case cannot be ascribed specifically to vortioxetine, aripiprazole, or their combination. These observations should therefore be considered hypothesis-generating and require further evaluation in systematic studies.

Adolescence is a developmental period marked by continued maturation of prefrontal–striatal networks involved in cognitive control, self-referential processing, and salience regulation (29). Alterations in these circuits during this developmental stage may intensify internally generated cognitive activity, contributing to persistent self-directed rumination and increased emotional reactivity (30). This developmental background may help explain why such clinical presentations often include prominent mood symptoms and diagnostic uncertainty in adolescents. Clinicians should carefully evaluate ego-dystonic internal dialogic intrusions in adolescents presenting with mood- or anxiety-dominant symptoms, as misinterpreting these experiences as psychotic phenomena may delay appropriate treatment.

## Limitations

This case report has several limitations. First, as a single-case observation, no causal inference can be made regarding pharmacological efficacy. The observed improvement occurred after sequential treatment changes, including discontinuation of sertraline, initiation of vortioxetine, and subsequent augmentation with aripiprazole; therefore, the relative contribution of each medication, psychotherapy, spontaneous symptom fluctuation, family support, and school re-engagement cannot be determined. Second, the initial treatment was not a fully optimized obsessive-compulsive disorder treatment trial. Sertraline was used at 100 mg/day for the initial depressive-anxiety presentation, but the dose was not further increased to the higher range commonly used in OCD. In addition, although structured cognitive behavioral therapy was provided, a manualized exposure and response prevention protocol was not completed, particularly because the obsessive-compulsive nature of the symptoms was not fully recognized at the early stage of treatment. This limits interpretation of the apparent limited response to the initial treatment strategy. Third, diagnostic and mechanistic assessments were limited. Although the diagnosis was supported by longitudinal clinical observation, mental status examination, and symptom severity ratings, structured psychosis-risk interviews, dissociation-specific assessments, and formal neurocognitive testing were not performed. No neuroimaging or neurocognitive measures were available to directly examine self-monitoring, cognitive inhibition, or salience-related mechanisms underlying the internal dialogue. Future studies should incorporate structured phenomenological assessment, standardized psychotherapy protocols, and longitudinal cognitive or neuroimaging measures to better characterize rumination-dominant obsessive-compulsive presentations with prominent internal dialogue in adolescents.

## Conclusion

This case highlights a less recognized clinical presentation of adolescent obsessive–compulsive disorder characterized by self-directed intrusive thoughts, secondary depressive symptoms, and marked functional impairment. The initial depression-dominant presentation obscured the obsessive nature of the condition, contributing to diagnostic complexity and delayed treatment. Treatment modification involving vortioxetine and subsequent low-dose aripiprazole augmentation was followed by symptom improvement and meaningful functional recovery, although causal attribution cannot be made from a single case. Early recognition of this clinical pattern and individualized treatment approaches may help reduce the risk of long-term academic and psychosocial difficulties in affected adolescents.

## Data Availability

The original contributions presented in the study are included in the article/supplementary material. Further inquiries can be directed to the corresponding author.
